# Relationship Between Gross Motor Skills and Inhibitory Control in Preschool Children: A Pilot Study

**DOI:** 10.3389/fnhum.2022.848230

**Published:** 2022-07-12

**Authors:** Jiajia Liu, Yiyan Li, Tang Zhou, Yanhua Lu, Menghao Sang, Longkai Li, Chunyi Fang, Wenwen Hu, Xiaojiao Sun, Minghui Quan, Jinyan Liu

**Affiliations:** ^1^School of Kinesiology, Shanghai University of Sport, Shanghai, China; ^2^Rehabilitation & Sports Medicine Research Institute of Zhejiang Province, Zhejiang Provincial People’s Hospital, People’s Hospital of Hangzhou Medical College, Hangzhou, China; ^3^Department of Physical Education, Institute of Disaster Prevention, Langfang, China; ^4^School of Physical Education and Sport Training, Shanghai University of Sport, Shanghai, China; ^5^Shanghai Frontiers Science Research Base of Exercise and Metabolic Health, Shanghai University of Sport, Shanghai, China; ^6^Department of Physical Education, Donghua University, Shanghai, China

**Keywords:** gross motor skills, locomotor skills, object control skills, inhibitory control, preschool children

## Abstract

**Purpose:**

Gross motor skills (GMS) and inhibitory control (IC) which are both development in preschool stage is significant for preschooler to healthy growth. However, the evidence of relationship between them in preschoolers are still insufficient, most of studies only focus on youth. Thus, the aim of this research is to examine the association between GMS and IC in preschool children.

**Methods:**

This cross-sectional study used baseline data from a previous intervention study of preschoolers conducted in 2018. GMS were assessed by using the Test for Gross Motor Development (2nd edition) in preschoolers, which includes two subtests of locomotor and object control skills. Total GMS is calculated from the sum of these two subtests. The Fish Flanker task was used to evaluate both accuracy and reaction time of IC. Multivariate linear regression models were established to analyze the relationships between GMS and IC.

**Results:**

A total of 123 preschool-age children (55 girls, 68 boys) were included in the final analysis. After adjusting for confounders, GMS (β = −8.27 ms, 95%CI: −14.2, −2.34), locomotor (β = −11.2 ms, 95%CI: −21.43, −0.97), and object control skills (β = −12.15 ms, 95%CI: −22.07, −2.23) were all negatively related with reaction time of IC.

**Conclusion:**

There was a significant negative correlation between gross motor skills and the reaction time of inhibitory control in preschool children. Further research is needed to verify this finding in prospective and experimental studies.

## Introduction

Executive functions (EF) are typically used to describe several top-down higher-order cognitive processes in the prefrontal cortex of the brain that include working memory, cognitive flexibility, and inhibitory control (Diamond, 2013). Collectively, these processes are important in one’s ability to plan, focus attention, remember, and juggle multiple tasks. Inhibitory control (IC) is an important role of executive function in young children as it relates to suppressing impulses, inappropriate behaviors, and dismissing goal-irrelevant stimuli in order to achieve desired goals (Brocki and Bohlin, 2004; Tiego et al., 2018). IC is divided into two subdomain Response Inhibition (the ability to inhibit one’s impulses to motor response) and Attentional Inhibition, also named interference inhibition (the ability to resist interference from unrelated stimuli) (Tiego et al., 2018). These subdomains often collective referred to as IC. A series of tasks have been used to measure IC in the past, such as Go/No-go, Stop-signal tasks, and Flanker task. Compared with other tasks, Flanker task is more suitable for preschoolers to understand and operate (Mcdermott et al., 2007). IC is important for preschool children, as it reflects their readiness to learn and adapt to complex situations. Preschoolers who fail to develop age-appropriate executive functions can present with low IC. Low IC is often reflected in children with attention deficit hyperactivity disorders (Thorell et al., 2009), difficulties in inhibiting their impulses by responding or reacting immediately to stimuli, an inability to express their thoughts easily (Diamond, 2016), a reduced attention span (Bocharov et al., 2021), and delayed motor development (i.e., a late transition from crawling to walking) (Mohd Nordin et al., 2021). IC also may be related in overeating and obesity (Batterink et al., 2010). Consequently, the promotion of safe and effective IC development activities in preschool children is of significance for their development and progress in educational pursuits.

Motor skills reflect the integration of a series of movements, gross motor skills (GMS) are one of primary parts of motor skills. GMS refers the large, force-producing muscles of limbs and the torso used to achieve a motor goal or task (Clark and Humphrey, 1994). GMS is developed in early life and are the building blocks for later complex motor skills (Barnett et al., 2016). GMS subtests include locomotor skills (LS; e.g., run, jump) and object control skills (OCS; e.g., kick a ball, catch a ball). These subtests are used to transport the body from one site to another and to project or receive objects, especially balls (Ulrich and Sanford, 2000). Evidence has shown that GMS is associated with physical activity and health-related physical fitness and that the development of GMS influences physical health status and motor performance across the lifespan from childhood into adulthood (Lubans et al., 2010).

The relationship between motor skills and executive functions originates from Piaget’s 1953 theory of cognitive development which posits the development of motor skills is influenced by a child’s interaction with their environment, thereby facilitating children’s cognitive development (Piaget and Cook, 1953). This view is supported by the 1993 research of Bushnell and Boudreau (1993) who further proposed that motor development is a prerequisite process for gaining and practicing other cognitive abilities, such as visual depth perception and haptic perception. Neuroimaging studies have shown that the association between GMS and IC is related to the co-activation of the cerebellum and the prefrontal cortex (Berman et al., 1995). Applied research has shown indirect associations between GMS and IC in developmental disorders, such as attention deficit hyperactivity disorders (Kaiser et al., 2015) and developmental coordination disorders (Rigoli et al., 2012). Several studies have investigated the associations between GMS and IC in children and/or adolescents (Roebers and Kauer, 2009; Geertsen et al., 2016), with primary interest in children with disabilities (Hartman et al., 2010; Michel et al., 2011; Schott and Holfelder, 2015). Meanwhile, in a systematic review of the associations between motor skills and cognitive function, including IC, in healthy children ages 4–16 years, Van der Fels et al. (2015) found either no association or a weak association between the variables among studies measuring this associations. Accordingly, the evidence is insufficient to prove the association between GMS and IC in healthy preschoolers. It is necessary to conduct additional studies. Preschoolers are at an important stage of developing GMS and IC (Garon et al., 2008; Tomaz et al., 2019). A favorable relationship between GMS and IC in healthy preschoolers can serve as a basis for future studies to understand the mechanisms for this relationship and to serve as a rationale for initiating intervention studies designed to improve GMS in preschool children.

The purpose of this study was to examine the relationships between GMS (total, LS, and OCS) and IC (IC-accuracy, IC-reaction time) scores obtained from the Fish Flanker task in healthy preschool children, ages 4–6 years. Based on a previous studies of GMS and IC in 5- and 6-year-old young children (Livesey et al., 2006), we hypothesize that GMS and IC scores obtained from standardized tests are negatively related.

## Materials and Methods

### Participants and Inclusion Criteria

The present study analyses cross-sectional, baseline data from The Influence and Mechanism of Aerobic Exercise on Preschool Children’s Executive Functions: A Randomized Controlled and Iconography Studies (Trial Registration: Chi CTR1900021552). The purpose of the above referenced study was to investigate the effects of an aerobic exercise intervention on the executive functions of preschool children. A total of 126 children aged 4–6 years (boys, 68; girls, 58) were recruited from preschools from four urban kindergartens in the Yangpu District of Shanghai, China. The preschools organized meetings with the students’ parents and/or guardians to explain the purpose and details of the study and to receive their assent for students to participate in the study. Inclusion criteria were as follows: (1) preschoolers aged 4–6 years; (2) good health with no contraindications to exercise, such as cardiovascular, neurological, or endocrine disease; and (3) receipt of informed assent forms signed and submitted by parents and/or guardians. The Ethics Committee of Shanghai University of Sport has approved this study (code: 2017023).

### Procedure and Measurements

#### Data Collection and Research Setting

All study activities were conducted from 9:00 to 11:00 am in participants’ kindergarten classes. The GMS and IC trials were implemented in a fixed sequence by a trained assessor and a trained assistant. To minimize the sources of measurement error, all tests were conducted by the same assessor. Parents and/or guardians completed demographic questionnaires developed for this study to collect information about the parent’s and/or guardian’s gender, age in years (y), maternal education (high school, college/associate degree, bachelor’s degree, master’s degree, doctoral degree), Annual per capita household income (< 9,000 RMB/per capita; 9,000 to 30,000 RMB/per capita; 30,001 to 100,000 RMB/per capita; and > 100,000 RMB/per capita) (1 RMB ≈ 0.16 US dollars), and the number of preschooler’s extracurricular classes taken outside of school (e.g., basketball, dancing, and badminton classes). Height in centimeters (cm) and weight in kilograms (kg) were measured on laboratory scales using procedures for preschool children identified in the National Physical Fitness Measurement Standards Manual (The General Administration of Sport of China, 2003). Body Mass Index (BMI, kg/m^2^) was computed as weight kg/height cm^2^ and the classification of BMI according to International Obesity Task Force (Cole et al., 2000).

#### Fish Flanker Task

**FIGURE 1:**
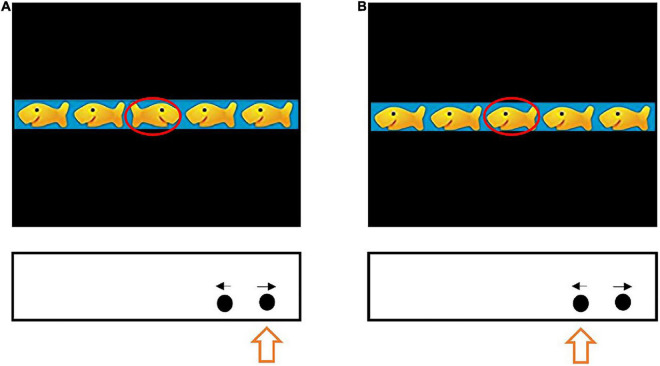
Fish flaker task.

A computerized Fish Flanker task was utilized to measure preschoolers’ IC and was implemented on E-Prime software (version 2.0, Psychological Software Tools, Pittsburgh, PA, United States). The Flanker paradigm has been described previously (Mcdermott et al., 2007). Briefly, the Fish Flanker task presents stimuli in a horizontal row of five fish with different orientations classified as a consistent condition (i.e., the direction of the fish is the same for all the fish) or as an inconsistent condition (i.e., more than one direction of the fish is observed). The goal of the task is to correctly identify if the direction of the middle fish (target fish). Standardized and age-appropriate instructions were used to convey the rules of the task to the participants. For example, children were asked “These fish are having fun. Please look at the fish in the middle that is hungry. If the middle fish is facing to the left, press the left button to feed it. If the middle fish is facing to the right, press the right button to feed it.” Figure 1 shows an image of the Fish Flanker task.

The Fish Flanker IC scores are presented as the time in milliseconds (ms) it takes to respond to the correct stimulus (IC-RT), presented as the average time across trials, and percent accuracy of responses (IC-ACC), computed as a percent (total number of trials divided by the number of correct trials). All participants completed the task as directed by experienced assessors in a quiet classroom setting. Participants were asked to respond as quickly as possible to the target fish on the screen independent of the fish to either side of the target fish (flanking fish) by pressing a button according to the direction of the target fish. A marker flashed for 500 ms at the beginning of the task as a cue that a target fish selection task would follow. The fish stimuli would be substitute by next fish stimuli after a response was made or until a response time more than 3000 ms. Tests were separated by 1500 ms. Prior to starting the trial, participants completed 20 practice tests with a goal to achieve 80% of the fish selected accurately. A total of 120 experimental trials were completed after the practice trials. An equal numbers of consistent and inconsistent trials were randomly intermixed and balanced such that all types of stimulus-flanker pairings were equally likely to occur. Participants were given a break of 5 min after their practice and after every 40 experimental trials. The participant’s performance for IC-ACC and IC-RT on the Fish Flanker tasks was recorded on the computerized E-Prime software (Martins et al., 2020). Data were considered invalid and were not included in the statistical analyses when participants pressed any key on the keyboard other than the directional < or > keys and when the reaction time was less than 200 ms or more than 3 s.

#### Test of Gross Motor Development Skills

Gross motor skills were measured by using the Test for Gross Motor Development (2nd edition, TGMD-2) which is globally viewed as the gold standard for GMS assessment with excellent test-retest reliability (Griffiths et al., 2018). The validity and reliability of TGMD-2 has been established for Chinese children (Li and Ma, 2007). The rater’s reliability, internal-consistency reliability and test-retest reliability of the TGMD-2 are *r* = 0.62 to 0.86, *r* = 0.72 to 0.89 and *r* = 0.87 to 0.94, respectively. Compared the final physical education scores, there are significant relationship between the final physical education scores and TGMD-2 score. TGMD-2 is comprised of two subtests (LS and OCS) with each subtest composed of six skills. The LS portion assesses running, galloping, hopping, leaping, jumping horizontally, and sliding. The OCS portion assesses striking a stationary ball, dribbling (bouncing) a stationary ball, kicking and catching a ball, throwing a ball overhand, and rolling a ball underhand. Prior to testing, assessors were trained on how to administer and evaluate each test performed according to the TGMD-2 training manual (see Supplementary Material). Also, videos were made to assist assessors in evaluating the skills. The inter-rater reliability of the assessors was high (ICC = 0.85, 95% CI = 0.73–0.92). Participants were tested in groups with six children per group. Participants waiting to perform their trials remained in the classroom and completed their regular lessons. Prior to the participants performing the tests, researchers demonstrated the correct form and procedures for each of the 12 skills. Participants performed each GMS skills’ test twice. Participants were scored according to the presence (score of 1) or absence (score of 0) of each criterion demonstrated during the test trials. Scores from each skill were added to obtain a total subtest score for the LS and the OCS portions of the total GMS score. The highest possible score for each LS and OCS subtest was 48. The two subtest scores were added to create the total GMS score with maximum score of 96.

### Statistical Analysis

Participant and parent and/or guardian characteristics are described as the mean and standard deviation (± SD) for continuous variables and the frequency (number and percentage) for categorical variables. Independent sample t-test were used to evaluate gender differences between the continuous and categorical variables, respectively.

A multivariate linear regression model was used to test the hypothesis that total GMS and subtests of LS, and OCS are significantly related to IC-ACC and IC-RT scores. Total GMS, and its’ subtests (LS, OCS) entered as independent variables, and IC (IC-ACC, IC-RT) entered as dependent variable in analysis. Two steps of analyses were performed. GMS scores entered as continuous variables in the first step and the GMS scores were categorized into Tertiles in the second step. In each analysis, data were computed as unadjusted (model 1) and adjusted analyses (model 2). In adjusted analyses, data were adjusted for potential confounders of age, BMI, gender, maternal education, household income, and the number of children’s extracurricular classes. In the first step of analysis, multivariate linear regression models with continuous independent and dependent variables are interpreted as follows: for each 1-point increment in the independent variable (total GMS, LS, OCS), there is an accompanying increase or decrease in the dependent variable (IC-ACC, IC-RT) with the amount of change determined by the direction and size of the beta coefficient.

In the second step of analysis, total GMS, LS, and OCS were divided into Tertiles with T1 containing the lowest scores and T3 containing the highest scores. The interpretation as follows: compared with the lowest scores T1, the dependent variable (IC-ACC, IC-RT) of T2 and/or T3 group improved or reduced. Unadjusted and adjusted multiple linear regression models tested the relationships between Tertiles of total GMS, LS, and OCS (T1 = referent group) with the continuous IC-ACC and IC-RT scores. Statistical analyses were performed with SPSS Statistics 22.0 (IBM, Armonk, NY, United States) and EmpowerStats software (www.empowerstats.com, X&Y solutions, Inc., Boston, MA, United States). *P* < 0.05 was accepted as statistically significant.

## Results

**FIGURE 2:**
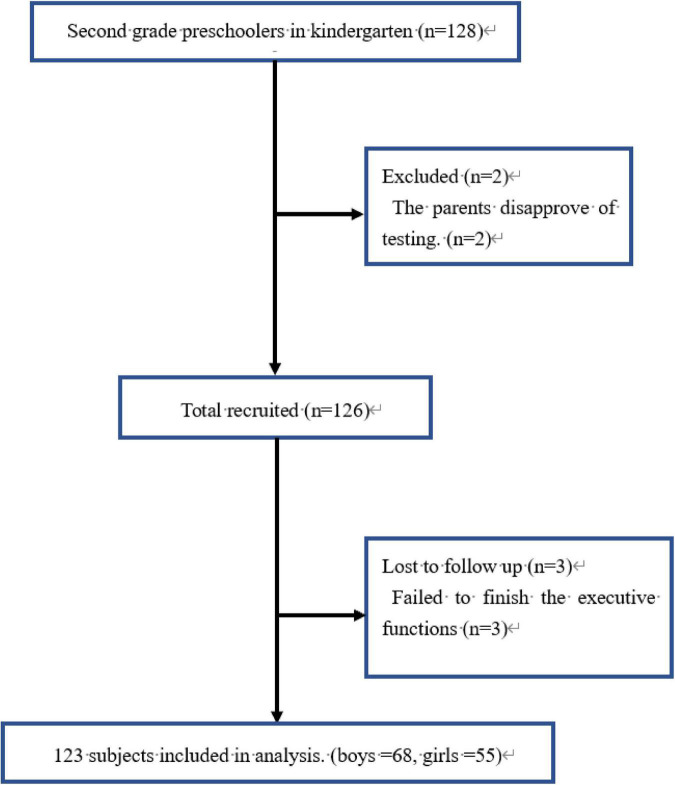
Flowchart of the subjects in the present study.

Among the 126 participants who completed the Fish Flanker task and TGMD-2 tests, data from 123 participants (68 boys and 55 girls; mean age, 4.89 ± 0.39 years) are included in the analysis (Figure 2). Three girls were excluded from data analysis due to their parents’ disapproval of testing subsequent to providing assent for their preschoolers to participate in the study. Characteristics of participants and parents and/or guardians, the GMS, and the IC scores are presented in Table 1. The majority of participants (83.06%) had a normal BMI according to the International Obesity Task Force (Cole et al., 2000). Height, weight, and BMI were higher in boys than girls (*P* < 0.01 to 0.05). LS scores for sliding were higher in girls than boys (*P* < 0.05) and OCS scores for striking and kicking skills were higher in boys than girls (*P* < 0.05). No significant differences by gender were observed for the remaining variables (*P* > 0.05).

**TABLE 1:** Characteristics of participants and parents and/or guardians and Gross Motor Skills and Inhibitory Control scores.

Variable	Girl (*n* = 55)	Boy (*n* = 68)	Total (*n* = 123)	*P* for sex
**Basic Demographic information**				
Age (years)	4.93 ± 0.42	4.86 ± 0.36	4.89 ± 0.39	0.32
Male sex, n (%)			68 (55.28%)	
**Anthropometric characteristics**				
Weight (kg)	18.58 ± 2.42	20.42 ± 3.39	19.60 ± 3.12	**< 0.01**
Height (cm)	109.83 ± 4.74	111.79 ± 4.90	110.91 ± 4.91	**0.03**
BMI (kg/m^2^)	15.36 ± 1.31	16.29 ± 2.11	15.88 ± 1.85	**< 0.01**
Normal	52 (94.55%)	53 (77.94%)	105 (85.37%)	
Overweight	3 (5.45%)	9 (13.24%)	12 (9.76%)	
Obesity	0 (0.00%)	6 (8.82%)	6 (4.88%)	
**Socioeconomic status**				
Household income (annual per capita RMB) n, (%)				0.65
< 9,000	5 (10.20%)	4 (6.06%)	9 (7.83%)	
9,000-30,000	7 (14.29%)	13 (19.70%)	20 (17.39%)	
30,001-100,000	18 (36.73%)	20 (30.30%)	38 (33.04%)	
>100,000	19 (38.78%)	29 (43.94%)	48 (41.74%)	
Maternal education, n (%)				0.69
High school	1 (2.04%)	2 (3.03%)	3 (2.61%)	
College/Associate’s degree	3 (6.12%)	8 (12.12%)	11 (9.57%)	
Bachelor’s degree	13 (26.53%)	12 (18.18%)	25 (21.74%)	
Master’s degree	26 (53.06%)	34 (51.52%)	60 (52.17%)	
Doctoral degree	6 (12.24%)	10 (15.38%)	16 (13.91%)	
Extracurricular class, n (%)				0.94
Yes, n (%)	33 (67.35%)	44 (66.67%)	77 (66.96%)	
No, n (%)	16 (32.65%)	22 (33.33%)	38 (33.04%)	
**Inhibitory control**				
Accuracy (ICC-ACC) (%)^a^	86.51 ± 14.76	86.06 ± 13.58	86.01 ± 14.05	0.38
Reaction time (ICC-RT) (ms)^b^	1236.34 ± 296.89	1233.16 ± 306.27	1240.31 ± 301.64	0.88
Total GMS (Score)	71.93 ± 10.09	71.85 ± 10.10	71.78 ± 9.95	0.84
Locomotor (Score)	38.18 ± 4.93	36.55 ± 6.59	37.28 ± 5.88	0.34
Run	6.95 ± 1.13	7.22 ± 0.99	7.10 ± 1.06	0.20
Gallop	6.85 ± 2.01	6.43 ± 2.56	6.62 ± 2.33	0.55
Hop	5.44 ± 2.17	4.81 ± 2.67	5.09 ± 2.47	0.23
Leap	5.38 ± 1.05	5.41 ± 1.05	5.40 ± 1.05	0.90
Horizontal Jump	6.40 ± 1.71	6.38 ± 1.79	6.39 ± 1.74	0.95
Slide	7.07 ± 1.44	6.37 ± 2.20	6.68 ± 1.92	**0.04**
Object-control (Score)	33.75 ± 7.09	35.30 ± 5.67	34.50 ± 6.26	0.17
Strike	7.38 ± 2.09	8.09 ± 1.71	7.77 ± 1.91	**0.04**
Stationary Dribble	5.49 ± 2.28	5.37 ± 2.29	5.42 ± 2.28	0.15
Catch	3.65 ± 1.60	3.65 ± 1.60	3.72 ± 1.51	0.92
Kick	5.93 ± 1.26	6.49 ± 1.25	6.24 ± 1.28	**0.02**
Overhand Throw	5.58 ± 2.07	6.19 ± 1.67	5.92 ± 1.88	0.07
Underhand Roll	5.33 ± 2.09	5.51 ± 1.88	5.43 ± 1.97	0.70

*^a^IC-ACC is computed by dividing the total number of tests performed by the number of tests with correct selections. ^b^IC-RT is the average reaction time of correct selections.*

*GMS, gross movement skill; BMI, Body Mass Index. The mean ± SD was reported for normal or non-normal distribution variables. The bold font is used to highlight significance level at P < 0.05.*

In the first step of analyses with total GMS, OCS, and LS scores analyzed as continuous variables, beta coefficients for total GMS, LS, and OCS scores were significantly and inversely related to IC-RT in model 1 (*P* < 0.05) and in model 2 (*P* < 0.05) (Table 2). None of the beta coefficients for the total GMS, LS, and OCS were related to IC-ACC (*P* > 0.05). For interpretations, after controlling for potential confounders in model 2, each 1-point increment in the total GMS, LS, and OCS scores resulted in a reduction in IC-RT by 8.27 ms (95% CI: −14.2, −2.34), 11.2 ms (95% CI: −21.43, −0.97), and 12.15 ms (95% CI: −22.07, −2.23), respectively.

**TABLE 2:** Associations between gross motor skill (Total GMS, LC, and OCS) presented as continuous scores and in Tertiles with inhibitory control scores (IC-ACC and IC-RT) on the Fish Flanker Test in preschool children ages 4-6 years (*N* = 123).

Variable	IC-ACC (accuracy),β (95%CI)		IC-RT (reaction time),β (95%CI)	
	Model 1	Model 2	Model 1	Model 2
***Total GMS (Score)***	0.24 (−0.01, 0.48)	0.09 (−0.16, 0.33)	−**6.08 (**−**11.35,**−**0.80)**	−**8.27 (**−**14.20,**−**2.34)**
***Total GMS tertile (Score)***				
T1 (42-70)	0 (Ref)	0 (Ref)	0 (Ref)	0 (Ref)
T2 (71-76)	**7.71 (1.73, 13.69)**	**6.47 (0.62, 12.33)**	−70.56 (−198.94, 57.81)	−109.35 (−250.13, 31.43)
T3 (77-92)	4.06 (−1.92, 10.04)	2.40 (−3.44, 8.23)	−**154.66 (**−**283.04,**−**26.29)**	−**216.98 (**−**357.39,**−**76.57)**
*P* for trend	0.092	0.25	**0.02**	**<0.01**
***Locomotor (Score)***	0.40 (−0.02, 0.83)	0.10 (−0.32, 0.53)	−**10.62 (**−**19.55,**−**1.70)**	−**11.20 (**−**21.43,**−**0.97)**
***Locomotor tertile (Score)***				
T1 (19-35)	0 (Ref)	0 (Ref)	0 (Ref)	0 (Ref)
T2 (36-40)	2.83 (−3.22, 8.87)	−1.37 (−7.46, 4.71)	−35.83 (−165.68, 94.01)	−18.85 (−166.68, 128.97)
T3 (41-46)	**6.18 (0.06, 12.29)**	1.27 (−4.80, 7.33)	−114.03 (−245.38, 17.31)	−116.97 (−264.37, 30.42)
*P* for trend	**0.05**	0.68	0.09	0.12
***Object-control (Score)***	0.24 (−0.16, 0.64)	0.14 (−0.28, 0.55)	−6.01 (−14.51, 2.49)	−**12.15 (**−**22.07,**−**2.23)**
***Object-control tertile (Score)***				
T1 (13-32)	0 (Ref)	0 (Ref)	0 (Ref)	0 (Ref)
T2 (33-37)	4.41 (−1.84, 10.66)	1.73 (−4.49, 7.96)	−58.86 (−193.34, 75.62)	−60.76 (−211.30, 89.78)
T3 (38-46)	0.74 (−5.41, 6.90)	−0.56 (−6.93, 5.81)	−62.67 (−195.07, 69.72	−147.55 (−301.63, 6.52)
*P* for trend	0.73	0.96	0.34	0.07

*Ref, Referent; GMS, gross movement skill; CI, confidence interval; The β values are standardized; The bold font is used to highlight significance level at P < 0.05; Model 1: Unadjusted; Model 2: Adjust for sex, age, BMI, maternal education, number of participants’ extracurricular classes, and per capita household income.*

In the second step of analysis with the total GMS, LC, and OCS scores divided into Tertiles, beta coefficients for T3 total GMS (models 1 and 2) and T3 LS (model 1) were negatively related to IC-RT (*P* < 0.05). Beta coefficients for T2 total GMS (model 2) and T3 LS (model 1) were positively related to IC-ACC (*P* < 0.05). For interpretations, T2 total GMS scores (model 1) were associated with a 6.18% (95% CI, 0.62, 12.29) higher IC-ACC score (*P* < 0.05), compared to T1 total GMS scores. T3 total GMS scores (model 2) resulted in a reduction in IC-RT by −216.98 ms (95% CI: −357.39, −76.57; *P*_trend_ < 0.01), compared to T1 total GMS scores.

## Discussion

This study aimed to investigate the relationships between GMS (total GMS, LS, and OCS) and IC (IC-ACC and IC-RT) in healthy preschool-age children ages 4-6 years. When analyzed as a continuous variable, total GMS, LS, and OCS scores showed significant and negative associations with IC-RT, indicating the preschool-age children with higher GMS scores spent less time selecting the correct answer on the Fish Flanker IC task than pre-school age children with lower GMS scores (*P* < 0.05). Total GMS scores divided into Tertiles showed a positive association between the T2 total GMS and IC-ACC scores and a negative association for T3 total GMS scores and IC-RT, indicating total GMS scores were related to speed and accuracy on the Fish Flanker task in preschool-age children (*P* < 0.05).

Few studies have evaluated the associations between motor skills and EF in children and adolescents. Mora-Gonzalez et al. (2019) showed that muscular fitness, speed agility, and cardiorespiratory fitness were related to EF in overweight and obese children aged 10.1 ± 1.1 years. Salcedo-Marin suggested that EF planning functions are mediated by various processes, such as cognitive processing speed and motor coordination in children and adolescents, ages 8-17 years, with attention deficit hyperactivity disorder (Salcedo-Marin et al., 2013). Ludyga et al. (2019) showed that OCS scores were inversely related to IC-RT in healthy, preadolescent children, ages 10-12 years. The present study showed similar associations in preschool children, ages 4-6 years, with total GMS, LS, and OCS scores negatively associated with IC-RT and Tertiles of total GMS T2 related to IC-ACC. While the results obtained from this study preclude identifying a causal association between GMS and IC, they are consistent with other studies observed in children and youth of different ages, health status, and developmental conditions.

Examination of studies investigating the associations between GMS and IC in preschoolers highlight the difficulty of comparing results obtained with varied methods and measurement scales. For example, in the present study, GMS was measured with the TGMD-2 and IC for task speed and accuracy was measured with the Fish Flanker task. Cook et al. (2019) measured GMS with the TGMD-2 and IC with the Go/No-Go tests. They reported positive and significant associations between LS (β = 0.2, *P* = 0.047) and OCS (β = 0.24, *P* = 0.024) scores and IC- ACC in preschoolers. Livesey et al. (2006) measured GMS with the Movement Assessment Battery for Children test and IC using the Modified stop-signal task. They observed a modest association between OCS and IC-ACC in preschoolers (*r* = 0.454, P < 0.05). In contrast, null relationships between GMS and IC-ACC were identified in the present study (*P* > 0.05). There are several reasons which may account for these inconsistent results. First, the psychological tasks used to assess IC differed in the types of IC assessed (Response Inhibition and Attention Inhibition) which are related to different neural networks in the brain (Verbruggen and Logan, 2008). The Go/No-go (Cook et al., 2019) and Stop-signal tasks (Livesey et al., 2006) are used to measure Response Inhibition which is related to the lateral and orbital prefrontal cortex. The Flanker task used in the present study and the Stroop test assess Attention Inhibition which is associated with anterior cingulate, dorsolateral prefrontal cortex and basal ganglia (Nigg, 2000). Second, the types of tests used to measure GMS skills differ between product- (e.g., jump height) and process-oriented (e.g., technique) outcomes. Livesey et al. (2006) used the Movement Assessment Battery for Children which is product-oriented whereas Cook (Cook et al., 2019) and the present study used the process-oriented TGMD-2 GMS test. Beside the results affected by the types of GMS and IC measures, socioeconomic status is an important factor between the development of GMS and IC processes in preschool-age children (Santos et al., 2008). The participants in the Cook et al. (2019) study were from a low- socioeconomic status population in South African settings, whereas the participants in the present study were from medium- to-high income Chinese families with a majority of their mothers (97.39%) having a college education. To establish consistency of results in future studies, researchers should strive to use the same measurement scales to assess GMS and IC.

### Possible Mechanism

Biological mechanisms for the associations between GMS and IC are based on evidence showing that areas of the brain involved in IC are closely related to motor pathways (Rae et al., 2015). Ludyga et al. (2021) reported an overlap in the neural networks between GMS and IC regions of the brain. The IC pathway involves the anterior cingulate, dorsolateral prefrontal cortex, and basal ganglia areas in the brain. Basal ganglia are highly involved in GMS as they contribute to inhibition of unnecessary movement and are important in mobilizing and coordinating body movements (Thach et al., 2000; Piek et al., 2004). In an animal study, Adkins et al. (2006) observed that motor training led to synaptogenesis synaptic potentiation, and reorganization of movement representations within the motor cortex. This is relevant as synapses affect the transmission efficiency of information (Cartling, 2002) and are related with cognitive development (Choi et al., 2018). Evidence shows that GMS shares common underlying processes with IC observed in sorting, monitoring, and planning tasks (Boxtel et al., 2001). Therefore, the skills which are acquired by preschoolers during the process of improving and mastering GMS may involve a transfer to the response processes of the brain related to IC. Booth et al. (2013) have reported that development of GMS facilitates physiological changes in the brain and improves the reaction ability of IC in preschoolers. Thus, IC-RT as a general measure of information processing speed may share a common underlying neurocognitive mechanism with GMS. The present results showing negative associations between GMS measures and IC-RT are consistent with the observation by Booth et al. (2013). Additional studies are needed to better understand the neurophysiology and mechanisms mediating the associations between GMS and IC.

### Applications

As it is necessary for preschoolers to pay attention to information, such as their location in space and the environment around them, it is important they learn how to restrain from attending to unrelated-goal behaviors in various settings, including during exercise. Having a fast cognitive reaction time means preschoolers spend a shortest time making a correct answer which reflects attention and is closely associated with executive functions (De Greeff et al., 2018). As evidence supports associations between GMS and IC, performing motor coordination tasks, and exercises that develop locomotor and object control skills should be encouraged in preschoolers. Activities that enhance motor coordination and GMS contribute to EF and IC by enhancing the efficiency of neurocognitive processing and the allocation of attentional resources in children’s immature brain states (Chang et al., 2013).

### Strengths and Limitations

To the best of our knowledge, few published studies (Van der Fels et al., 2015) of the relationship between GMS and IC have focused on healthy preschool children and those that have used GMS and IC tasks that differ from those used in the present study. Existing studies measure the accuracy of IC tasks, but none have measured the speed of completing an IC task. A strength of the present study is that it used valid and reliable GMS and IC tests to determine the association between total GMS, LS, and OCS abilities with EF IC-ACC and IC-RT in preschoolers with a wide range of motor skills.

There were also limitations associated with the present study, including a relatively small sample size of 123 preschool-aged children and the cross-sectional design. The cross-sectional design prevented causal relations from being drawn between GMS and IC. Furthermore, the participants in this study were Chinese and mostly from medium- or high-income communities, and a majority of their mothers received higher education. This corresponded with the participants receiving high IC-ACC scores (mean ± SD: 86.01 ± 14.05), indicative that the trial was very easy for them. Additional studies with similar participants are needed to confirm these results.

## Conclusion

In summary, the results of this study demonstrated a negative relationship between total GMS components of locomotor and object control skills with the executive functions IC subsets of reaction time, and to a lesser extent, accuracy in responding to the Fish Flanker task in preschool children. The results provide further support for the hypothesis that GMS are related to specific aspects of IC and they highlight the importance of teaching motor skills to preschoolers. As the biological mechanisms between GMS and IC are not fully understood, research showing the variables may share common underlying biological processes is promising and calls for further studies designed to understand how motor skills are related to cognitive process of EF, and in particular, IC-RT. Cross-sectional, prospective, and experimental studies in preschoolers are encouraged to use the same measurement scales to confirm the results GMS and IC obtained in this study.

## Data Availability Statement

The raw data supporting the conclusions of this article will be made available by the authors, without undue reservation.

## Ethics Statement

The studies involving human participants were reviewed and approved by the Ethics Committee of Shanghai University of Sport (code: 2017023). Written informed consent to participate in this study was provided by the participants’ legal guardian/next of kin.

## Author Contributions

JJL: conceptualization, validation, formal analysis, and writing—original draft preparation. MQ: methodology and resources. WH, CF, and LL: software. YYL, MS, YHL, WH, CF, LL, XS, and TZ: investigation. JJL and MQ: data curation. XS and MQ: writing—review and editing. JYL: visualization, supervision, project administration, and funding acquisition. All authors have read and agreed to the published version of the manuscript.

## Conflict of Interest

The authors declare that the research was conducted in the absence of any commercial or financial relationships that could be construed as a potential conflict of interest.

## Publisher’s Note

All claims expressed in this article are solely those of the authors and do not necessarily represent those of their affiliated organizations, or those of the publisher, the editors and the reviewers. Any product that may be evaluated in this article, or claim that may be made by its manufacturer, is not guaranteed or endorsed by the publisher.

## Abbreviations

EF: executive function
GMS: gross motor skill
IC: inhibitory control
LS: locomotor skills
OCS: object control skills
IC-ACC: the accuracy of inhibitory control
IC-RT: the reaction time of inhibitory control
TGMD-2: the Test for Gross Motor Development – 2nd edition
BMI: body mass index.
