# Supplementary material for: Relationship Between Gross Motor Skills and Inhibitory Control in Preschool Children: A Pilot Study
Source: Front Hum Neurosci. 2022 Jul 12;16:848230. doi: 10.3389/fnhum.2022.848230 (PMC9314641; doi:10.3389/fnhum.2022.848230)

Supplementary Figure 1. Locomotor Subtest

| Skill | Materials |  | Performance Criterial | Trial 1 | Trails 2 | Score |
| --- | --- | --- | --- | --- | --- | --- |
| 1. Run | 60 feet of clear space and two cones. | Place tow cones 50 feet apart. Make sure there is at least 8 to 10 feet of space beyond the second cone for a safe stopping distance. Tell the child to run as fast as he or she can form one cone to the other when you say “Go”. Repeat a second trail. | 1. Arms move in opposition to legs. elbows bent. |  |  |  |
|  |  |  | 1. Brief period where both feet are off the ground. |  |  |  |
|  |  |  | 1. Narrow foot placement landing on heel or toe (i.e., flat footed). |  |  |  |
|  |  |  | 1. Nonsupport leg bent approximately 90 degrees (i.e., close to buttocks). |  |  |  |
| Skill Score | | | | | |  |
| 2. Gallop | 25 feet of clear space, and tape or two cones. | Mark off a distance of 25 feet with two cones or tape. Tell the child to gallop from one cone to the other. Repeat a second trial by galloping bake to the original cone. | Arms bent and lifted to waist level at takeoff. |  |  |  |
|  |  |  | A step forward with the lead foot followed by a step with the trailing foot to a position adjacent to or behind the lead foot. |  |  |  |
|  |  |  | Brief period when both feet are off the floor. |  |  |  |
|  |  |  | Maintains a rhythmic pattern for four consecutive gallops. |  |  |  |
| Skill Score | | | | | |  |
| 3. Hop | Minimum of 15 feet of clear space. | Tell the child to hop three times on his or her preferred foot (established before testing) and then three times on the other foot. Repeat a second trail. | 1. Nonsupport leg swings forward in pendular fashion to produce force. |  |  |  |
|  |  |  | 1. Foot of nonsupport leg remains behind body. |  |  |  |
|  |  |  | 1. Arms flexed and swing forward to produce force. |  |  |  |
|  |  |  | 1. Takes off and lands three consecutive times on preferred foot. |  |  |  |
|  |  |  | 1. Takes off and lands three consecutive times on nonpreferred foot. |  |  |  |
| Skill Score | | | | | |  |

Continued

| Skill | Materials |  | Performance Criterial | Trial 1 | Trails 2 | Score |
| --- | --- | --- | --- | --- | --- | --- |
| 4. Leap | A minimum of 20 feet of clear space, a beanbag and tape. | Place a beanbag on the floor. Attach a piece of tape on the floor so it is parallel to and 10 feet away from the beanbag. Have the child stand on the tape and run up and leap over the beanbag. Repeat a second trial. | 1. Take off on one foot and land on the opposite foot. |  |  |  |
|  |  |  | 1. A period where both feet are off the ground longer than running. |  |  |  |
|  |  |  | 1. Forward reach with the arm opposite the lead foot. |  |  |  |
| Skill Score | | | | | |  |
| 5. Horizontal Jump | A minimum of 10 feet of clear space and tape. | Mark off a starting line on the floor. Have the child start behind the line. Tell child to jump as far as he or she can. Repeat a second trial. | 1. Preparatory movement includes flexion of both knees with arms extended behind body. |  |  |  |
|  |  |  | 1. Arms extend forcefully forward and upward reaching full extension above the head. |  |  |  |
|  |  |  | 1. Take off and land on both feet simultaneously. |  |  |  |
|  |  |  | 1. Arms are thrust downward during landing. |  |  |  |
| Skill Score | | | | | |  |
| 6. Slide | A minimum of 25 feet of clear space, a straight line, and two cones | Place the cones 25 feet apart on top of a line on the floor. Tell the child to slide from one cone to the other and back. Repeat a second trial. | 1. Body turned sideways so shoulders are aligned with the line on the floor. |  |  |  |
|  |  |  | 1. A step sideways with lead foot followed by a slide of the trailing foot to a point next to the lead foot. |  |  |  |
|  |  |  | 1. A minimum of four continuous step-slide cycles to the right. |  |  |  |
|  |  |  | 1. A minimum of four continuous step-slide cycles to the right. |  |  |  |
| Skill Score | | | | | |  |
| Locomotor Subtest Raw Score (sum of the 6 skill scores) | | | | | |  |

Supplementary Figure2.Object Control Subtest

| Skill | Materials |  | Performance Criterial | Trial 1 | Trails 2 | Score |
| --- | --- | --- | --- | --- | --- | --- |
| 1. Striking a Stationary Ball | A 4-inch light weight ball, a plastic bat, and a batting tee. | Place the ball on the batting tee at the child’s belt level. Tell the child to hit the ball hard. Repeat a second trial. | 1. Dominant hand grips bat above nondominant hand. |  |  |  |
|  |  |  | 1. Nonpreferred side of body faces the imaginary tosser with feet parallel. |  |  |  |
|  |  |  | 1. Hip and shoulder rotation during swing. |  |  |  |
|  |  |  | 1. Transfers body weight to front foot. |  |  |  |
|  |  |  | 1. Bat contacts ball. |  |  |  |
| Skill Score | | | | | |  |
| 2. Stationary Dribble. | An 8-10-inch playground ball for children ages 3 to 5; a basketball for children ages 6 to 10; and a flat, hard surface. | Tell the child to dribble the ball four times without moving his or her feet, using one hand and then stop by catching the ball. Repeat a second trial. | 1. Contacts ball with one hand at about belt level. |  |  |  |
|  |  |  | 1. Pushes ball with fingertips (not a slap). |  |  |  |
|  |  |  | 1. Ball contacts surface in front of or to the outside of foot on the preferred side. |  |  |  |
|  |  |  | 1. Maintains control of ball for four consecutive bounces without having to move the feet to retrieve it. |  |  |  |
| Skill Score | | | | | |  |
| 3. Catch | Minimum of 15 feet of clear space. | Tell the child to hop three times on his or her preferred foot (established before testing) and then three times on the other foot. Repeat a second trail. | 1. Preparation phase where hands are in front of the body and elbows are flexed. |  |  |  |
|  |  |  | 1. Arms extend while reaching for the ball as it arrives. |  |  |  |
|  |  |  | 1. Ball is caught by hands only. |  |  |  |
| Skill Score | | | | | |  |

Continued

| Skill | Materials |  | Performance Criterial | Trial 1 | Trails 2 | Score |
| --- | --- | --- | --- | --- | --- | --- |
| 4. Kick | An 8 – to 10-inch plastic, playground, or soccer ball; a beanbag; 30 feet of clear space; and tape. | Mark off one line 30 feet away from a wall and another line 20 feet from the wall. Place the ball on top of the beanbag on the line nearest the wall. Tell the child to stand on the other line. Tell the child to run up and kick the ball hard toward the wall. Repeat a second trail. | 1. Rapid continuous approach to the ball. |  |  |  |
|  |  |  | 1. An elongated stride or leap immediately prior to ball contact. |  |  |  |
|  |  |  | 1. Nonkicking foot placed even with or slightly in back of the ball. |  |  |  |
|  |  |  | 1. Kicks ball with instep of preferred foot (shoelaces) or toe. |  |  |  |
| Skill Score | | | | | |  |
| 5. Overhand Throw | A tennis ball, a wall, tape, and 20 feet of clear space. | Attach a piece of tape on the floor 20 feet from a wall. Have the child stand behind the 20 foot line facing the wall. Tell the child to throe the call hard at the wall. Repeat a second trail. | 1. Windup is initiated with downward movement of hand/arm. |  |  |  |
|  |  |  | 1. Rotates hip and shoulders to point where the nonthrowing side faces the wall. |  |  |  |
|  |  |  | 1. Weight is transferred by stepping with the foot opposite the throwing hand. |  |  |  |
|  |  |  | 1. Follow-through beyond ball release diagonally across the body toward the nonpreferred side. |  |  |  |
| Skill Score | | | | | |  |
| 6. Underhand Roll.  6. Underhand Roll. | A tennis ball for children ages 3 to 6; a softball for children ages 7 to 10 two cones; tape; and 25 feet of clear space. | Place the two cones against a wall so they are 4feet apart. Attach a piece of tape on the floor 20 feet from the wall. Tell the child to roll the ball hard so that it goes between the cones. Repeat a second trail. | 1. Preferred hand swings down and back, reaching behind the trunk while faces cones. |  |  |  |
|  |  |  | 1. Strides forward with foot opposite the preferred hand toward the cones. |  |  |  |
|  |  |  | 1. Bends knees to lower body. |  |  |  |
|  |  |  | 1. Release ball close to the floor so ball does not bounce more than 4 inches high. |  |  |  |
|  |  |  | 1. Preferred hand swings down and back, reaching behind the trunk while faces cones. |  |  |  |
| Skill Score | | | | | |  |
| Object Control Subtest Raw Score (sum of the 6 skill scores) | | | | | |  |


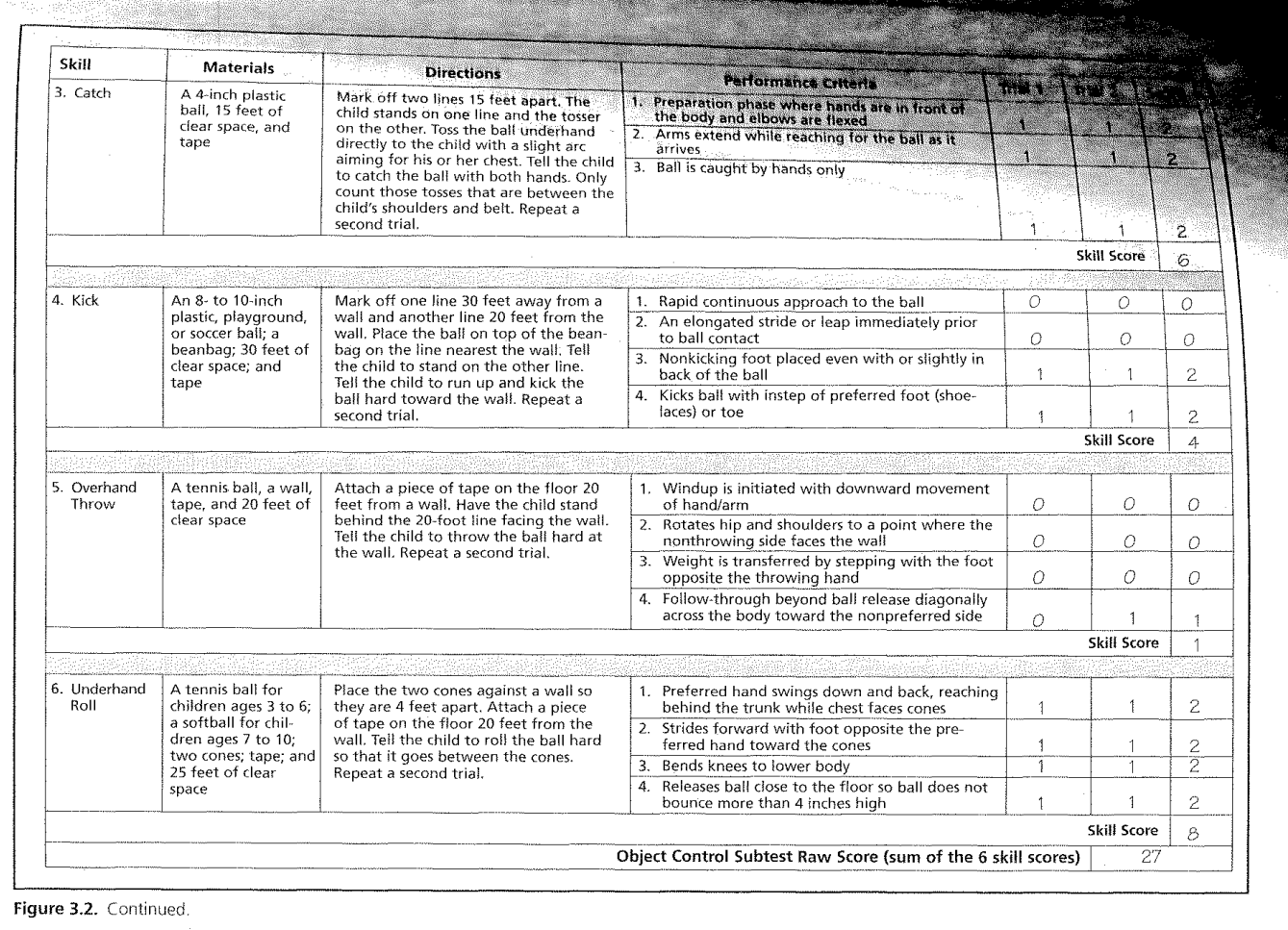

Supplement: Supplementary file 1 [file Data_Sheet_1.docx]
